# Implementing electronic patient record systems (EPRs) into England’s acute, mental health and community care trusts: a mixed methods study

**DOI:** 10.1186/s12911-015-0204-0

**Published:** 2015-10-14

**Authors:** Arabella Clarke, Joy Adamson, Laura Sheard, Paul Cairns, Ian Watt, John Wright

**Affiliations:** Department of Health Sciences, University of York, York, UK; Bradford Institute for Health Research, Bradford Royal Infirmary, Bradford, UK; Department of Computer Sciences, University of York, York, UK

## Abstract

**Background:**

Our aim was to explore the approaches to and the challenges and benefits of implementing Electronic Patient Record systems (EPRs) into NHS acute, mental health and community care hospitals throughout England.

**Methods:**

A mixed methods approach was adopted that comprised an online survey (*n* = 59) and semi-structured telephone interviews (*n* = 8) with chief information officers (or heads of EPR projects) at NHS trusts throughout England. Survey analysis was descriptive, whilst the qualitative interviews were analysed thematically.

**Results:**

A range of devices and approaches to implementing EPRs were described with 32 % of survey respondents utilising a best of breed approach. Interviewees’ perceived and expected benefits of implementing an EPR included efficiency, availability and accessibility of clinical information, and patient safety. Key challenges to EPR implementation were securing clinician involvement, difficulties posed by government and national policy and limited availability of financial and human resources.

**Conclusions:**

There was no single approach regarding the approaches taken to implementing EPRs among participating English NHS trusts, with various benefits and challenges cited. Policymakers and researchers need to provide clearer guidance for trusts at various stages of implementation ensuring intelligence is shared across England’s NHS trusts.

**Electronic supplementary material:**

The online version of this article (doi:10.1186/s12911-015-0204-0) contains supplementary material, which is available to authorized users.

## Background

Political pressure for hospitals in the English National Health Service (NHS) to implement Electronic Patient Record systems (EPRs) has been mounting over recent years, notably through Department of Health (DoH) initiatives such as, ‘Safer hospitals, Safer wards; achieving an integrated digital care record’ [1] and the Secretary of State’s call for a paperless NHS by 2018 [2]. What is more, previous failures of national Information Technology (IT) policy such as the national programme for IT in the NHS (NPfIT) [3] add to the pressure on NHS hospitals to ‘get it right’. National NHS IT policies are often costly with a lack of demonstrable benefits. For example, the ‘Safer hospitals, Safer wards: achieving an integrated digital care record’ [1] initiative has cost the NHS £500mn since its publication in 2013, with £60mn of the first instalment being unallocated due to trusts failure to demonstrate a return of investment [4].

**Fig. 1:**
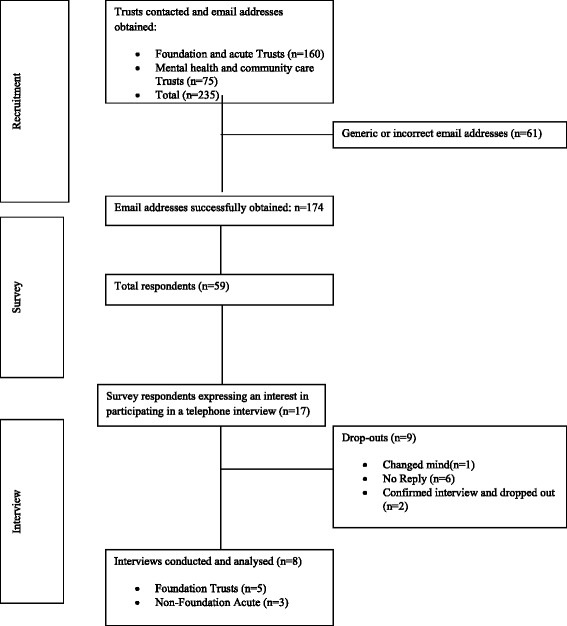
Participant flow during the study

The political and financial support from NHS England and DoH for trusts to implement these systems implies there is a strong evidence base supporting the idea that EPRs can improve health outcomes and quality of care. In reality, literature, supporting these claims is either predominately from the U.S. whose health service has different economic, organisational and structural foundations from the UK, or from reports listing ‘potential’ future benefits, rather than empirical evidence [5–7]. The literature is focussed on proposed benefits of implementing these systems once they are fully ‘up and running’ and so little evidence currently exists which reports benefits that have been realised and the challenges of implementing these systems into NHS organisations.

Furthermore, despite the political and financial implications of implementing EPRs, there is a lack of empirical evidence surrounding EPR implementation in the UK. A recent systematic review [8] examined Electronic Health Record (EHR) implementation, which included EPR literature showing U.S. hospitals to be at different stages of implementation with varying levels of EPR functionality [9]. The EPR literature also found a number of challenges and disadvantages to EPR implementation including; reduced doctor productivity, technological issues such as software design [9, 10] and information sharing and confidentiality [8]. Whilst the literature included in the systematic review [8] was primarily from the US there were some studies from the UK but these focussed on EHR implementation [11, 12]. There is a degree of uncertainty as to the differences between EHRs and EPRs. Historically an EHR was a longitudinal record of a patient’s health care from cradle to grave which combines information regarding patient contact with primary care and periodic care held within EPRs [13]. An EPR (or EMRs as they are known in the U.S.) was therefore a separate system to an EHR, and is for the purposes of this paper defined as a record containing episodic care typically by one institution relating to patient information such as personal details, diagnosis and treatment [13]. However over time, these terms have been used interchangeably, making the dissemination and use of literature in this area difficult as it is unclear what type of system is being investigated. This lack of guidance from research arguably makes achieving government targets such as ‘a paperless NHS by 2018’ more challenging. Therefore, this study explores the approaches to and benefits and challenges of implementing EPRs into English acute, mental health and community care NHS trusts.

## Methods

A mixed methods approach was taken involving an online survey and qualitative interviews with Chief Information Officers (CIOs). The online survey was distributed between October and November 2013 via email and was a census of all 235 acute, community care and mental health trusts in England. As there is no centralised record held by the Department of Health (DoH) of contact details for CIOs (or heads of EPR) and following DoH guidance a list of all NHS acute, community care and mental health trusts switchboard telephone numbers were obtained via the NHS Choices website. All 235 trusts were then contacted to obtain email addresses for their CIO or equivalent individual. However, a lack of consistency in job title and in some cases inability or unwillingness to provide this information meant that email addresses for communications, human resources or IT departments were often obtained rather than for a specific individual. When generic email addresses were obtained, an email including a link to the survey was sent indicating that the survey should be sent to the CIO or equivalent person in that trust. Reminder emails, which included the link to the survey, were sent to all participants (unless they expressed disinterest) 2 weeks after the initial email was sent and again in the final week of the one month period for which the survey was available.

The survey (Additional file 1) which was developed using SurveyMonkey [14] comprised fixed questions relating to trust demographics (trust type and geographical location) and the hardware and solution strategies used to implement EPRs. Hardware strategies are devices through which an EPR may be accessed i.e. computers on wheels (COWs) or fixed desktops. Solution strategies are the approaches or ‘architecture’ trusts are using to implement EPR systems (Additional file 2). The survey was informed and piloted with Chief Information Officers (CIOs) at two NHS trusts in the North of England. Survey questions were analysed using SPSS for windows version 19 and descriptive analysis was undertaken.

For the qualitative interviews, individuals were recruited using convenience sampling of those who indicated they would be happy to participate in a telephone interview at the end of the survey. A topic guide (Additional file 3) provided a framework for the semi-structured interviews and was informed and piloted with the same CIOs as the survey. Interviews aimed to provide more detailed exploration of the approaches to implementation (hardware and solution strategies) to complement the survey data as well as to gain an understanding of the benefits and challenges of implementing an EPR. Telephone interviews were conducted between November 2013 and January 2014 and lasted between 20 and 40 min. Interviews were transcribed verbatim with all participants assigned a unique ID code. Interviews were analysed using the five stages of thematic analysis as outlined by Braun and Clarke [15]; transcription, familiarisation, coding, theme development and data reporting. Coding and theme development was deductive with themes driven by the topic guide. Members of the research group were consulted throughout the analysis of interviews and theme and code development to enhance plausibility of the findings. Reflective notes [16] were taken following each interview and throughout the analysis with methodological, analytical and personal reflections noted and considered during the interpretation of the study’s findings.

After the initial analysis had been completed for both the qualitative and quantitative material the data were integrated. A list of themes from both the preliminary analyses was created and where both types of data were available for the same theme this has been presented together. For some themes only quantitative or qualitative data were available (i.e. the benefits of implementing an EPR) and so these themes are presented separately. During the process of data integration some themes were re-named or re-fined. For example, the theme hardware and solution strategies was re-structured and re-named so as to combine both quantitative and qualitative data reflecting the approaches and systems used by trusts implementing EPRs. Quotations were selected from the interviews that were illustrative of the point being [15].

The sociotechnical approach was used to inform data integration and to facilitate the interpretation of study findings. The approach challenges the notion that IT system implementations fail solely due to technical reasons and can be used to understand why an implementation strategy may be successful in one organisation yet not in another [17]. The use of the approach in this study is based upon the work of Greenhalgh et al. [18] who used the approach to evaluate electronic summary care records in England. During their evaluation Greenhalgh et al., [18] suggest that when evaluating technology programs quantitative and qualitative methods should be used to study the macro (National policy, wider social norms) meso (organisational processes) and micro (particular experiences of patients and professionals) level sociotechnical influences [18]. As the approach is predominately used to understand why IT implementations fail, the three levels of sociotechnical thinking have provided a useful means of organising and understanding the various challenges affecting the implementation of EPRs in this study.

Ethical approval was granted by the University of York, Health Sciences Research Governance Committee (11/10/2013). Informed written consent was obtained for the interviews, for the survey implicit consent was taken from those who completed the questionnaire.

## Results

### Participants

Fifty nine of the 235 trusts invited to participate in the survey responded (25 %). However, assuming that the response rate was differential with 63 email addresses being either generic departmental email addresses or incorrect an adjusted response rate of 59 participants from 174 correct emails would ensue (34 %). A flow diagram illustrating the number of trusts recruited and included in the survey and interviews are shown in Fig. 1. The majority of survey and interview respondents were implementing an EPR, *n* = 47 and *n* = 6 respectively. Survey respondents represented a range of locations: North England (*n* = 16); East England and the Midlands (*n* = 3) and London and South England (*n* = 15) with 25 respondents not specifying their location. Survey and interview respondents also represented a range of NHS trust types including: Foundation Trust (n = 26, (n = 3); Non Foundation Acute Trust (n = 13), (n = 5), Mental Health and Community Care Trust (n = 11) and Combined (*n* = 9). Trusts within the combined category were those that indicated that they were Mental Health, Community Care and Foundation Trust. Demographic characteristics for survey and interview respondents are displayed in Additional file 4.

### The approaches and systems used by trusts to implement EPRs

There was no consensus among survey respondents as to the type of systems used to deliver EPRs with 37 different systems cited. Additional file 5 displays the most frequently (2 or more trusts) used electronic systems adopted.

Hardware strategies mainly included fixed desktops (*n* = 55, 93.2 %), with trusts also incorporating COWs (*n* = 39, 66 %), handheld PCs (*n* = 47, 79.7 %), tablets (*n* = 41, 69.5 %) and smartphones (*n* = 38, 64.4 %). Additionally, 58 % (*n* = 34) of trusts were using five or more types of devices to deliver their EPRs.

Interviewees commented on their experiences of using these devices, with positive experiences of laptops and mobile devices reported and the benefits of these devices including; mobility, flexibility and data capturing. In relation to tablets, these were not being used extensively, or were being trialled with concerns expressed over confidentiality, security, maturity; battery life and compatibility with existing software. Contrasting experiences of COWs were reported with the devices used mainly for their mobility and to enable the use of computers at the bedside:

Respondent 020401: *so people love the COWs*… *that tends to be the most*…*popular item here* (*laughs*) *mostly because they round with them*…*and they round with*… *medical students and residents and they pull up on the screen everything that they need to see on a patient chart and then do what they need to do*…*I love COWs I think everywhere I have worked people have liked them* (*Respondent 020401*, .

Respondent 010301: *our experience with both sets of COWs has been very negative in that there is a huge problem with the battery life and the speed of access and everything on the computers and so the COWs and there also big*…*difficult to move around and so they tend to even though there mobile they tend to be moved to one bit of a ward and or just left there*.

A range of approaches to EPR implementation were cited by survey respondents, with the most popular approach being ‘best of breed’ (32 % *n* = 19) (Additional file 2). Furthermore, of the 28 % (*n* = 17) of participants using a combination of solution strategies, nine of the 11 combinations included a best of breed approach. Interviewees were also largely utilising a best of breed approach, with a variety of reasons including that trusts cannot afford or are unable to identify a lack of suitable megasuite system. Despite the popularity of the best of breed approach, a number of interviewees raised concerns regarding the layout and usability of best of breed systems which was seen as complex and inconsistent. When comparisons were made between megasuite and best of breed approaches, benefits and challenges were attributed to both. Furthermore, the trusts that had adopted a megasuite approach were better resourced, had substantial clinician involvement within their projects and tended to have higher digital maturity.

Respondent 082404: *when we were doing the procurement we did look at whether it*’*s best of breed or whether it*’*s a megasuite I*’*m with you now*…*actually there*’*s quite a lot of published evidence to say that best of breed is not necessarily the best solution and the interfaces can become quite complex*…*and we have experienced some really bad interfacing problems and actually as it turns out even with what you call a megasuite there are still interfaces*.

### Benefits of implementing EPRs

Perceived benefits were centred around patient safety, efficiency and information availability and accessibility, with both future benefits and benefits that had already been realised mentioned. Only two interviewees referred to benefit realisation plans or business cases. EPRs were viewed to have the potential for a host of patient safety benefits centred around e-prescribing and advanced decision support including reduced prescribing errors, and prompting for best practice respectively. Whether patient safety benefits were considered to have been realised depended on a trust’s digital maturity, with more mature trusts citing benefits such as the ability to better monitor deteriorating patients. In contrast, less mature trusts reported often failing to see benefits to the extent they expected. Trusts maturity was determined in a number of ways, including whether interviewees directly mentioned digital maturity indexes such as HIMSS and their position on these scales and also at what stage of implementing EPRs they were at for instance, pre-procurement, procurement, implementation, full EPR implemented:

Respondent 061803: *to accrue better and more substantial benefits electronic pharmacy having been the most obvious example where that will start to stop having situations of repeat prescribing and when there*’*s a necessary appropriate control of prescribing which is contraindicated*.

Efficiency benefits were expected as a result of EPRs, enabling speedier flows of information, data entry and completion of tasks such as discharges. In addition to time saving, not having paper reduced prescribing costs from e-prescribing and reduced diagnostic tests were perceived to bring the potential for cost saving efficiencies. Efficiency benefits that had been realised were mainly from the reduced storage of notes and administrative staff, as well as having all clinical information accessible:

Respondent 051601: *we*’*re*…*able to turn around things like pathology and radiology reports much faster*.

Interviewees also suggested that there will be, and in some instances have been, benefits from EPRs improving the quality, availability and accessibility of information. These benefits are expected to result from having all patient information in one place, whilst also enabling the use and sharing of information within and across health and social care organisations.

Respondent 030608: *the ability to share that sort of information and turn that information into working knowledge that we use again for new patients*…*I mean that’s what it’s all about really*

### Challenges to implementing EPRs

The majority of interviewees reported engaging clinicians in trust EPR projects to be a challenge. This was attributed to clinicians having varied IT skills or willingness to be involved. Interviewees also mentioned the difficulty of managing clinicians’ expectations in terms of what can be achieved within projects. Clinicians were perceived to expect projects to have a clear point of completion, whereas in reality participants explained how the ‘shifting landscape of technology’ creates new technological possibilities and means projects are rarely ‘complete’. The increased societal use of technology was also perceived to have raised clinicians expectations of what technology should be available at work, due to the sophistication of technology that individuals are used to at home to complete a range of daily tasks:

Respondent 010301: *actually you have a huge variation in those who are keen to use IT and those that are not keen to use IT and so engagement with the staff and getting them to understand the importance of utilising the technology that we have has been a big challenge…that’s probably one of the biggest challenges we have.*

For a number but not all interviewees, a lack of finance and other resources such as time or trusts current technological capability was a challenge to implementing EPRs. More specifically, there was talk surrounding financial instability and constraints preventing trusts from achieving their EPR ambitions. The different financial and resourcing situations of NHS trusts was also perceived to have created an ‘unequal playing field’ exacerbated by national policy which sets the same aims for trusts irrespective of their digital maturity or financial and resourcing capabilities leaving some trusts to play ‘catch up’:

Respondent *061803: if we’d had greater continuity of management and more resources available to us in the first place instead of having to fight for each and every project, whilst we had two or three reasonable years the last two years have been fairly dire in terms of resource because of the situation we are in financially.*

Government and national IT policy was cited as a challenge, with participants explaining how they feel as though they are ‘battling’ against ‘political milestones’ that are enforced upon them, despite these milestones not always viewed as clinically relevant. There was also tension surrounding The National Program for IT (NPfIT) [3] with participants still considering the program to be hindering the innovation and implementation of EPRs, to the extent that the NHS is perceived to be behind other countries such as the U.S. Whilst the program was considered by participants initially to be a good idea, it is now viewed as a lost opportunity that is still hindering innovation and development of EPRs to the extent that the UK and NHS are behind other countries.

Respondent 071212: *lack of foresight because actually when a lot of these things come out of number 10 or wherever they come out of there is people on the ground that are going oh no and yet somebody still thinks this is a good idea…I mean who thinks the friends and family test is a good idea [laughs] collecting that and reporting it by ward it’s a terrible idea but we have to waste our time doing it….so the reality is that we waste a lot of our time doing things that are completely useless and add no value to the clinical service whatsoever so there’s something that I would change*.

A further challenge was perceived to be quantifying benefits of implementing an EPR or showing a return of investment. Reasons for this include the multifaceted and often subjective nature of benefits that are often not realised for some time:

Respondent 051601: *like any sort of other large business the focus is very much on return of investment… and return of investment is traditionally financial models…how you…do a financial business case to stop one child’s been in a safeguarding sort of incident is again very difficult to model up…and it becomes a bit like an insurance policy…a business case providing an insurance policy […]I paid 200 pounds a month for my car insurance never claim it but I still think I need it (laughs) so it’s when times are hard to have those sorts of systems that support those risk management insurance stuff is very difficult.*

It was also suggested that software and technology is behind trusts ambitions as despite wanting clinicians to have access to records through one device, there is currently no device that can provide this or that is suitable for all clinical requirements. Ultimately resulting in clinicians carrying or accessing multiple devices. Moreover, the lack of universally accepted or out of the box solution or approach to implementing EPRs is an additional technological challenge when implementing EPRs:

Respondent 051601: *one of the real challenges we’ve got is people and we want clinicians to be mobile so as to have access to information they need from wherever they are but what we find is software vendors or solution providers are a little bit behind on that curve.*

A summary of the challenges to EPR implementation using the three levels of sociotechnical influence; meso, macro and micro can be found in Table 1.

**Table 1:** Macro, meso and micro factors affecting the implementation of EPRs

Macro		Meso		Micro	
Factor	Description	Factor	Description	Factor	Description
*National policy, and government influence*	Impact of NPfIT and impact of government targets that are not clinically focussed.	*Showing a return of investment*	Trusts are failing to show a return of investment. Business cases were rarely mentioned.	*Clinicians IT knowledge and willingness to be involved in IT projects*	Whether clinicians are engaged and/or adopt EPR systems can depend upon their IT skills and willingness to be involved.
*Implementation strategies/solution strategies*	There is no single best approach to implementing EPRs available with trusts utilising a range of devices, systems and strategies when implementing EPRs.	*Finance and resources*	Trusts have varying finance and resources available despite being required to reach the same government targets.	*Clinician Involvement in EPR projects*	At an organisational level, whether trusts involve clinicians and how they involve them.
*Technology and hardware available*	The technology available is not mature enough to meet clinical needs and to enable one device to be used for all tasks.			*Managing Clinician expectations*	Clinicians often expect EPR projects to be completed or have high expectations of what can be achieved within the scope of EPR projects.

## Discussion

This study highlights that there is no consensus as to the approaches being used to implement EPRs into English NHS trusts. Despite a best of breed approach being the most common solution strategy among participants, this only represented 32 % of survey respondents with interviewees citing pros and cons to the approach. Furthermore, of the 59 participating trusts 37 different systems providers of EPRs were used. Whilst the study respondents provide no real insight into the ‘best’ approach to EPRs the study does provide the first account of the various approaches being used and the associated pros and cons to these approaches and devices. For instance, comparisons were made between best of breed and megasuite approaches with participants criticising the usability of a best of breed approach, but acknowledging that there is a lack of suitable megasuite systems available for use within the NHS. Furthermore, this is the first empirical English study to explore the approaches to and benefits and challenges of implementing an EPR into an English NHS trust and may therefore be useful to NHS trusts of varied digital maturity and stages of implementation.

This study provides English empirical evidence of both realised and expected benefits of implementing EPRs. The findings support those of a recent qualitative interview study that reported, ‘anytime anywhere access to patient information’ and ‘time and human resource related efficiency savings to be benefits experienced by clinicians of an EPR within the NHS [19]. However, despite both UK policy documents [1] and the academic literature surrounding EPRs stating that EPRs are implemented as they have the potential to improve the quality and safety of healthcare [13], there remains little empirical evidence on the realised benefits of EPR systems within the NHS. Whilst this is perhaps a reflection of the stage of implementation at which NHS hospitals are at in comparison to other countries, the necessity of establishing UK benefits at all stages of EPR implementation should not be underestimated. This is of particular importance given that benefits literature is used in the formulation of trusts business cases and applications for funding which at present are reliant on US literature that would not be necessarily applicable to the NHS.

The lack of guidance and evidence surrounding the implementation of EPRs has been acknowledged by previous policies such as safer hospitals safer wards: integrating a digital care record [1]. This is exacerbated by the failure of policy to adopt either a top down approach or decentralisation, causing confusion as to which aspects of EPR implementation is the responsibility of NHS organisations or that of central government. NHS England are starting to address this issue through the Clinical Digital Maturity Index (CDMI) [20], however it is important that the CDMI is clear and provides enough detail to help trusts progress to digital record keeping in a realistic and unambiguous fashion. Moreover, there is a risk of the CDMI crudely classifying trusts as being high or low in terms of maturity, which could cause those at the lower end of the spectrum from being disenfranchised and the ‘unequal playing field’ from widening.

The use of both quantitative and qualitative methods underpinned by sociotechnical thinking builds on the recommendation by Greenhalgh et al., [18] that a technology programme should be studied at the meso, macro and micro level and both quantitatively and qualitatively in order to understand complex change. The approach also provides a useful way of organising and interpreting the various social and technical influences affecting the implementation of EPRs. Whilst the majority of challenges to implementation identified in this study were at the macro level, it would seem important that all three levels are considered and that they are addressed at national, organisational and individual level. Equal weight should therefore be given to sociological and technological issues, something which informatics literature has received criticism for previously [17].

The main limitation for the study was the small number of survey respondents. This was in part due to the lack of centralised record of CIOs available from the DoH or consistency of job title for those responsible for trusts EPR projects, making recruitment a particular challenge. However of the sample obtained varying levels of EPR maturity and a range of trust types and locations were represented (Additional file 4). In relation to the qualitative interviews, despite 8 being a relatively small number given the range of participants and trusts interviewed, this sample may be adequate to meet the aims of the study. Furthermore, due to the complexity of implementing EPRs and the variety of benefits, challenges and approaches being used by trusts, a higher response rate of both the survey and interviews would not take away from this picture. Therefore, whilst the full range of viewpoints may not be represented this is unlikely to have impacted on the study’s findings.

Another finding from the study was the lack of centralised record of CIOs, or indeed consistency of job title. Given the lack of a universal approach to the implementation of EPRs and the recent acknowledgement of the need for NHS trusts to share insights, knowledge and experience with one another as they move towards EPRs by NHS England [1]; a centralised record either nationally or regionally would help to facilitate intelligence sharing and assisting one another in the implementation and development of EPR systems.

Lastly, the aim of this paper was not to create an overly negative portrayal of EPRs, as these systems will undoubtedly bring benefits to the NHS in the future. However, our findings suggest that for the individuals who are implementing these systems, the challenges currently outweigh the benefits. Given the amount of literature that focuses on the potential benefits of these systems, which is based on limited empirical evidence, it is important that the challenges associated with implementing EPRs are also documented.

## Conclusions

With the rising pressure on NHS trusts to implement EPRs, there is a need for policymakers to provide better guidance as to the best routes to implementation. To achieve this, policymakers need more UK evidence in larger samples, especially surrounding the approaches, challenges and benefits of implementing EPRs in NHS organisation. Furthermore, greater sharing of lessons among NHS organisations should be encouraged and facilitated by Academic Health Science Networks and the CDMI if NHS trusts are expected to move up the digital maturity ladder.

## Additional files

Additional file 1:
**Survey questions.** (PDF 42 kb) Additional file 2:
**Solution strategies adopted by participants.** The approaches or solution strategies (e.g. best of breed) adopted by participating trusts to implement EPRs. (PDF 86 kb) Additional file 3:
**Topic guide for semi-structured interviews.** Topic guide used for semi-structured interviews with Chief Information Officers. (PDF 45 kb) Additional file 4:
**Demographic characteristics.** Demographic characteristics for survey and interview respondents (EPR status, trust type, location and interviewees job title). (PDF 14 kb) Additional file 5:
**The different EPR systems being implemented by trusts in the sample.** The different EPR systems (i.e. RiO, Millenium) being implemented by participating trusts. (PDF 138 kb)
