# Supplementary material for: Implementing electronic patient record systems (EPRs) into England’s acute, mental health and community care trusts: a mixed methods study
Source: BMC Med Inform Decis Mak. 2015 Oct 14;15:85. doi: 10.1186/s12911-015-0204-0 (PMC4607108; doi:10.1186/s12911-015-0204-0)
Supplement: Additional file 1: — Survey questions. (PDF 42 kb) [file 12911_2015_204_MOESM1_ESM.pdf]

Additional file 1: Survey

1. Which of the following options best describes your trust? (Please tick all that apply)

- [ ] Foundation trusts
- [ ] Acute trusts
- [ ] Mental health trusts
- [ ] Community Care trusts
- [ ] Yorkshire and the Humber
- [ ] London
- [ ] West Midlands
- [ ] South West
- [ ] South East
- [ ] South Central
- [ ] North West
- [ ] North East
- [ ] East England
- [ ] East Midlands

2. Do you currently have any form of Electronic Patient Record?

- ○ Yes
- ○ No

3. What systems do you currently use to provide the EPR?

4. Will this system allow you to go paperless by 2018?

- ○ Yes
- ○ No

If No what is your plan for achieving this goal?

5. What functions does your EPR currently include?

- [ ] Clinical Decision Support
- [ ] Results
- [ ] Ordering Drugs
- [ ] Words
- [ ] Numeric

Other (please specify)

6. When will your trusts EPR be authorised as a definitive record, so the paper record will no longer be required or used?

7. What is or will be your solution strategy for your EPR system?

- Best of Breed
- Write it yourself
- Someone else to write it
- Megasuite system
- Clinical EPR, separate interfaced PAS

Other (please specify)

8. Do you see your EPR as being:

- Only for your organisation with an interface required to outside systems
- Part of a multi organisation shared system

Other (please specify)

9. What is your hardware strategy for enabling EPR and how useful have you found it? Please rate 1-5 where 1 = Useless, 5= Very Useful

	Not Used	1	2	3	4	5
COW	*What is your hardware strategy for enabling EPR and how useful have you found it? Please rate 1-5 where 1 = Useless, 5= Very Useful COW Not Used	COW 1	COW 2	COW 3	COW 4	COW 5
Handheld PCs	Handheld PCs Not Used	Handheld PCs 1	Handheld PCs 2	Handheld PCs 3	Handheld PCs 4	Handheld PCs 5
Fixed desktops	Fixed desktops Not Used	Fixed desktops 1	Fixed desktops 2	Fixed desktops 3	Fixed desktops 4	Fixed desktops 5
iOS devices	iOS	iOS	iOS	iOS	iOS	iOS

	Not Used	1	2	3	4	5
	devices Not Used	devices 1	devices 2	devices 3	devices 4	devices 5
Android devices	Android devices Not Used	Android devices 1	Android devices 2	Android devices 3	Android devices 4	Android devices 5
PDAs	PDAs Not Used	PDAs 1	PDAs 2	PDAs 3	PDAs 4	PDAs 5
Tablets	Tablets Not Used	Tablets 1	Tablets 2	Tablets 3	Tablets 4	Tablets 5
Smartphones	Smartphones Not Used	Smartphones 1	Smartphones 2	Smartphones 3	Smartphones 4	Smartphones 5

Comment field

10. Would you be happy to take part in a follow up telephone interview?

Yes

No

If Yes please provide an email address below
