# Supplementary material for: Implementing electronic patient record systems (EPRs) into England’s acute, mental health and community care trusts: a mixed methods study
Source: BMC Med Inform Decis Mak. 2015 Oct 14;15:85. doi: 10.1186/s12911-015-0204-0 (PMC4607108; doi:10.1186/s12911-015-0204-0)
Supplement: Additional file 2: — Solution strategies adopted by participants. The approaches or solution strategies (e.g. best of breed) adopted by participating trusts to implement EPRs. (PDF 86 kb) [file 12911_2015_204_MOESM2_ESM.pdf]

Additional file 2 Solution strategies adopted by survey participants

Solution Strategy	Description	Frequency (%)
Best of Breed	Multiple and different departmental systems are typically implemented gradually and incrementally. Individual modules or departmental systems (i.e. clinical decision support, e-prescribing) are chosen based on the needs of the clinical specialty with the replacement of one module not affecting or disrupting other specialties . Interoperability is therefore a large factor and refers to making the different systems work together, this is usually achieved through an integration engine.	19 (32)
Megasuite	A term used to describe the EPR systems provided by suppliers such as Cerner (Millennium) or Meditech (EPIC). These systems normally contain the full range of clinical and non-clinical EPR functionality that a hospital requires in the form of a single integrated solution rather than via a suite of separate systems interfaced to each other via an integration engine as in a best of breed approach.	10 (16)
Write it yourself	Providers or trusts that choose to build their own EPR solution rather than procuring one from a commercial supplier. The provider may source the development capacity in-house or contract it out to an external development resource. For example, trusts who have a team of programmers supporting in-house EPR.	3 (5)
In Development	Participants who indicated that their solution strategy was in development or undecided.	4 (6)
Combined approach	Best of Breed and write it yourself (n=3)<br><br>Best of breed and clinical EPR with separate interfaced PAS (n=3)<br><br>Best of breed and someone else to write it (n=2)<br><br>Best of breed, clinical EPR separate interfaced PAS someone else to write it,	17 (28)

	write it yourself (n=1)<br><br>Best of breed, write it yourself, clinical EPR with separate interfaced PAS (n=1)<br><br>Best of breed write it yourself, someone else to write it (n=1)<br><br>Best of breed and own developments (n=1)<br><br>Best of breed someone else to write it and clinical EPR with separate interfaced PAS (n=1)<br><br>Primary system, some best of breed some procured some own developments (n=1)<br><br>Someone else to write it and clinical EPR with separate interfaced PAS (n=1)<br><br>Write it yourself and other unspecified solutions (n=1)<br><br>Unspecified combination (n=1)	
Other	An integrated EPR and Integrated system with community	2 (3)
