# Supplementary material for: Implementing electronic patient record systems (EPRs) into England’s acute, mental health and community care trusts: a mixed methods study
Source: BMC Med Inform Decis Mak. 2015 Oct 14;15:85. doi: 10.1186/s12911-015-0204-0 (PMC4607108; doi:10.1186/s12911-015-0204-0)
Supplement: Additional file 3: — Topic guide for semi-structured interviews. Topic guide used for semi-structured interviews with Chief Information Officers. (PDF 45 kb) [file 12911_2015_204_MOESM3_ESM.pdf]

Topic Guide: For use with Chief Information Officers

At the beginning of all interviews:

1. Introduce self
2. Just as a reminder. The interview will be used to supplement data collected during the survey which you have previously completed. The study aims to understand and explore the EPR systems being implemented across NHS trusts throughout England with a specific focus on the different systems being implemented and the different strategies being used to implement the EPR.
3. This interview will be audio recorded, direct quotations may be used and published but all identifiable information will be removed and kept confidential. In order to ensure this all participants will receive pseudonyms.
4. Data will be used as part of my PhD thesis and may also be used in any resulting publications or presentations at conferences. Additionally the results of the study will be fed back to the CIO and mobile device user group at Bradford. However in any write up and dissemination of the results all interview data will be anonymous and all data will be stored confidentially on a password protected computer in a locked room.

Interview:

1. Please could you tell me about your involvement in setting up your trusts EPR system

2 What does a paperless hospital mean to you?

3. What is your Trusts vision in terms of EPR and being paperless and how is it defined?

4. Please could you outline your trusts processes for setting up the EPR system
    - the drivers for the project,
    - the team involved,
    - whether there was any clinician involvement?

5. According to the survey your solution strategy was ‘**best of breed** with a hardware strategy that includes **COW, handeld PCs fixed desktops, IOS, androidsTablets smartphones**. Please could you tell me about how they are used in your trust and why you decided upon these methods?

6. What explicit benefits and outcomes are you expecting to achieve as a result of your hospitals vision, and EPR system (including non-cash related benefits)?

7. Have you realised any of these benefits yet if so which ones?

8. Of the benefits you are yet to realise how are you hoping to achieve them?

9. What challenges have you faced so far throughout the design and implementation of your IT strategy?

-How could things have been different/how could you have improved that

End the interview:

1. So firstly I would just like to thank you for your time today.
2. Just to reiterate the interview data may be published and will be used in a Phd thesis. However all data will be stored confidentially and all data will be made anonymous.
3. If you have any questions
4. If you have any questions after the interview please feel free to contact me via email
5. Also if you would like a summary of the research findings once they have been analysed and produced please let me know and I can send these to you via email.
