# Supplementary material for: Implementing electronic patient record systems (EPRs) into England’s acute, mental health and community care trusts: a mixed methods study
Source: BMC Med Inform Decis Mak. 2015 Oct 14;15:85. doi: 10.1186/s12911-015-0204-0 (PMC4607108; doi:10.1186/s12911-015-0204-0)
Supplement: Additional file 4: — Demographic characteristics. Demographic characteristics for survey and interview respondents (EPR status, trust type, location and interviewees job title). (PDF 14 kb) [file 12911_2015_204_MOESM4_ESM.pdf]

Additional file 4 Demographic characteristics of survey and interview respondents

	Frequency	
	**Survey Respondents**	**Interviewees Respondents**
	**N (%)**	**N**
**EPR Status**		
**Yes**	47 (79.7)	6
**No**	12 (20.3)	2
**Trust Type**		
Foundation Trust	26 (44.1)	3
Non Foundation Acute Trust	13 (22)	5
Mental Health/Community Care Trust	11 (18.6)	0
Combined	9 (15.2)	0
**Trust Location**		
North England	16 (27.1)	4
East England and the Midlands	3 (5.1)	1
London and South England	15 (25.4)	3
Unspecified Location	25 (42.3)	-
**Job Title**		
Clinical Information Officer and clinical safety officer	-	1
Director/associate director of IT	-	2
Director of IM&T and deputy director of IM&T	-	2
Director of PAS system	-	1
Director of Informatics	-	1
Program Director	-	1
