# Supplementary material for: Implementing electronic patient record systems (EPRs) into England’s acute, mental health and community care trusts: a mixed methods study
Source: BMC Med Inform Decis Mak. 2015 Oct 14;15:85. doi: 10.1186/s12911-015-0204-0 (PMC4607108; doi:10.1186/s12911-015-0204-0)
Supplement: Additional file 5: — The different EPR systems being implemented by trusts in the sample. The different EPR systems (i.e. RiO, Millenium) being implemented by participating trusts. (PDF 138 kb) [file 12911_2015_204_MOESM5_ESM.pdf]

Additional file 5 The EPR systems being implemented by trusts in the sample

Type of system	Frequency	Description
Best of Breed	9	See table 2
In House Developments	9	See table 2
RiO (RiO mental health)	6	An EPR software solution for recording and documenting health care services. Predominately used in mental health and community health settings (22).
Sunquest ICE	5	An integrated clinical environment providing a range of products mainly associated with results and reporting. Other features include; discharge, wristband and label printing (23).
Systm One (Systm One community)	4	Comprises a range of modules (hospital, GP, child health) used together or in isolation. The 'hospital module' includes; PAS, bed management, e-prescribing, e-discharge, A&E and clinical record viewer. (24)
KAINOS Evolve	4	An EPR enabling the creation, capture and handling of medical records. Extra features include; e-discharge, patient manager (planning and monitoring clinical activity), timeline (an at a glance patient history) and living forms (to monitor patient progress) (25).
Civica Paris	4 (2 Civica Windip)	EPR and care management system for monitoring and managing community and social care. WinDip is an electronic workflow and document management system complementary to the EPR (26)
Ascribe	3	A range of solutions which include PAS, order communications, e-prescribing, scheduling, e-handover (27).
Systm C (Medway, Careplus, Liquidlogic)	3	A range of solutions to health and social care such as: Medway EPR, Medway Maternity, Careplus (child health records solution) Liquidlogic (child or adult social care system) (28).
IPM	3	Integrated patient management solution which allows the whole patient journey to be tracked within a single system (29).
Millenium	2	An EPR allowing real time access to patient results and clinical information. Features include; handover, real time dashboards, real time bed management, prescribing, clinical decision support (30).
Graphnet	2	Can be used as either a shared record viewer across organisations or a single EPR in an acute trust. Provides immediate access to up-to-date patient information, assessments, data collection, workflow and notifications across organisations (31).
PACS	2	A system enabling images such as X-rays to be stored electronically and viewed on screens so information can be accessed and compared to previous images (32).
Meditech	2	An EHR comprising a suite of integrated applications including EPR and PAS which include components such as; order communications, A&E management, theatre management, document management and

		results reporting (33)
No response	7	
